# Safety and Effectiveness of Denileukin Diftitox‐cxdl in Patients With Relapsed/Refractory Peripheral T‐Cell Lymphoma and Cutaneous T‐Cell Lymphoma: An All‐Case Post‐Marketing Study in Japan

**DOI:** 10.1002/cnr2.70641

**Published:** 2026-08-16

**Authors:** Tatsuro Jo, Kota Matsumoto, Mika Ishii, Koji Izutsu

**Affiliations:** ^1^ Department of Hematology Japanese Red Cross Nagasaki Genbaku Hospital Nagasaki Japan; ^2^ Eisai Co., Ltd. Tokyo Japan; ^3^ Department of Hematology National Cancer Center Hospital Tokyo Japan

**Keywords:** cutaneous T‐cell lymphoma, denileukin diftitox, peripheral T‐cell lymphoma, post‐marketing study

## Abstract

**Introduction:**

Denileukin diftitox (DD)‐cxdl, a fusion protein comprising diphtheria toxin fragments and human interleukin‐2, is a treatment for peripheral T‐cell lymphoma (PTCL) and cutaneous T‐cell lymphoma (CTCL). Previous trials have enrolled a limited number of patients, and there is a need to evaluate DD‐cxdl in a more diverse population, including those with different ages and treatment histories.

**Methods:**

This observational post‐marketing study (19 May 2021 to 1 February 2024) was conducted in over 200 institutions in Japan to evaluate the safety and effectiveness of DD‐cxdl in patients with relapsed or refractory PTCL and CTCL (NCT05137847; jRCT2031220196). Patient characteristics, status of drug administration, adverse events (AEs), adverse drug reactions (ADRs), and effectiveness were assessed. Capillary leak syndrome was specified as an AE of special interest.

**Results:**

Data from 104 patients were analyzed (PTCL: 85; CTCL: 19), including 57 male patients (54.8%). The median age was 74 (range: 20–88) years. The median number of prior chemotherapy lines was 3.5 (1.0–10.0), and approximately half (55.8%) of the patients discontinued treatment during cycle 1. Most patients experienced AEs (94.2%) and ADRs (84.6%), and the most common serious ADR was capillary leak syndrome (22.1%). Effectiveness could be assessed in 80/104 patients. The overall objective response rate was 16.3% (95% confidence interval: 8.9, 26.2), and in patients with PTCL and CTCL it was 15.4% (7.6, 26.5) and 20.0% (4.3, 48.1), respectively. Four patients (6.2%) with PTCL achieved a complete response.

**Conclusions:**

This is the first observational study to evaluate the safety and effectiveness of DD‐cxdl in Japanese patients with relapsed or refractory PTCL and CTCL in daily clinical practice. No new safety concerns were identified. Clinicians should remain aware of the risk of capillary leak syndrome.

**Trail Registration:** ClinicalTrials.gov identifier: NCT05137847 and Japan Registry of Clinical Trials identifier: jRCT2031220196.

## 1 Introduction

Peripheral T‐cell lymphoma (PTCL) and cutaneous T‐cell lymphoma (CTCL) are rare and heterogeneous forms of mature T‐cell neoplasms among non‐Hodgkin lymphomas (NHLs) [1, 2]. PTCL accounts for 12%–15% of NHLs and is characterized by an aggressive clinical course, frequent relapses, and the eventual development of refractory disease [1, 3]. CTCL often presents as an indolent condition and refers to a heterogeneous group of NHLs with a poor prognosis in advanced‐stage disease [4, 5].

In Japan, cyclophosphamide, doxorubicin, vincristine sulfate, and prednisolone (CHOP) combination therapy [6, 7] is widely used for the treatment of both PTCL and CTCL. Furthermore, several drugs have been approved for the treatment of relapsed or refractory T‐cell NHLs, including pralatrexate, romidepsin, forodesine, tucidinostat, mogamulizumab, brentuximab vedotin, valemetostat, and darinaparsin for PTCL; and vorinostat, mogamulizumab, brentuximab vedotin, and bexarotene for CTCL. However, many patients with PTCL or CTCL do not adequately respond to treatment, and the objective response rate (ORR) for these agents is 25%–34% [8, 9, 10, 11, 12]. Hence, there is a need for more treatment options for patients with relapsed or refractory PTCL and CTCL.

Denileukin diftitox (DD) is a recombinant cytotoxic fusion protein comprised of the amino acid sequences for diphtheria toxin fragments A and B, and human interleukin‐2 (IL‐2) [13, 14]. It was developed by Seragen (now Ligand Pharmaceuticals) in the United States. A therapy containing DD as the active ingredient, E7272 [15, 16], was approved in the United States in October 2008 following Phase III trials conducted in patients with persistent or recurrent IL‐2 receptor‐α (CD25)‐positive CTCL [17, 18].

E7777, which has the same amino acid sequence as E7272, was developed in the United States using an improved manufacturing process and has an improved purity and an increased percentage of the active monomer [19]. A Phase II trial of E7777 in Japanese patients with PTCL and CTCL reported an ORR of 36.1% (95% confidence interval [CI]: 20.8, 53.8) [20]. The most common adverse events (AEs) were generally manageable, but the authors noted that the occurrence of the potentially fatal capillary leak syndrome is an AE that warrants closer investigation [20, 21].

Following the Phase I and II trials of E7777 [20, 22], the drug was approved for use in patients with PTCL and CTCL in Japan in March 2021. A Phase III trial of E7777 was conducted in the United States and Australia and reported an efficacy and safety profile consistent with a previous trial of DD‐cxdl [23]. Thus, E7777 was approved for the treatment of adult patients with relapsed/refractory Stage I–III CTCL after ≥ 1 previous systemic therapy in the United States in August 2024 [24]. Currently, therapies containing DD‐cxdl are often used as second‐line or later treatment for PTCL and CTCL [25]. DD‐cxdl is approved for the treatment of PTCL and CTCL in Japan, and only for CTCL outside Japan.

Although clinical trials provide critical evidence regarding efficacy and safety, the eligibility criteria can exclude certain patients who would otherwise be encountered in daily clinical practice. Furthermore, both the Phase I and II trials of E7777 had limited sample sizes, with 13 patients in the Phase I trial [22] and 37 patients (17 with PTCL, 19 with CTCL, and 1 with another type of lymphoma) in the Phase II trial [20].

Thus, there remains a need to evaluate the safety and effectiveness of DD‐cxdl in a more diverse real‐world population of patients, including those with different ages and treatment histories. To address this, we conducted a post‐marketing study to determine the safety and effectiveness of DD‐cxdl in Japanese patients with relapsed or refractory PTCL and CTCL in daily clinical practice. We also investigated factors that may impact the safety of DD‐cxdl.

## 2 Methods

### 2.1 Study Design and Patients

This retrospective observational post‐marketing study was conducted in over 200 institutions in Japan between 19 May 2021 and 1 February 2024. All patients who received DD‐cxdl in Japan were included in this study. The registration period was between 19 May 2021 and 30 September 2022. The observation period was planned for a minimum of 24 weeks, but varied depending on treatment duration. For patients who discontinued treatment before 24 weeks, data were collected up to the time of discontinuation. Conversely, for those who continued treatment beyond 24 weeks, follow‐up data were collected for the extended period. The study data were collected using an Electronic Data Capture system. Physicians entered the reason for treatment discontinuation and the evaluation up to the time of discontinuation. Discontinuation was defined as either when administration of the treatment ceased or when observation was no longer possible because the patient did not attend hospital visits.

This study was conducted according to the Japanese Good Post‐Marketing Study Practice (GPSP; the Japanese Ministry of Health, Labour and Welfare Ordinance No. 171, 2004) [26]. According to the Guidance for the Japanese Ethical Guidelines for Medical and Biological Research Involving Human Subjects, a post‐marketing study conducted in accordance with the GPSP is outside the scope of these Ethical Guidelines [27]. Therefore, informed consent and institutional review board approval under these Ethical Guidelines were not required. Nevertheless, this study was centrally approved by the Clinical Research Advisory Board of Eisai Co. Ltd. on December 17, 2019. Patient data were anonymized, ensuring confidentiality and privacy.

### 2.2 Treatment

Patients received DD‐cxdl at a dose of 9 μg/kg, once daily, by intravenous infusion over 1 h for 5 days, with a 16‐day gap between treatments (21 days in total per cycle) [28]. Up to eight cycles were administered, and the dose was reduced, if required, at the physician's discretion depending on the patient's condition.

### 2.3 Study Assessments

The following assessments were performed: patient background characteristics, status of drug administration, AEs, adverse drug reactions (ADRs), and effectiveness.

AEs were defined by the Common Terminology Criteria for Adverse Events (CTCAE) v4.0 Japanese Translation Japan Clinical Oncology Group (JCOG) Edition, and severity was determined according to the criteria in the CTCAE. Items not listed in these criteria were evaluated per the five severity grades according to the severity judgment criteria for items not listed in the CTCAE v4.0 Japanese Translation JCOG Edition. Serious AEs were defined as those resulting in death, life‐threatening, requiring hospitalization or prolongation of hospitalization, persistent or significant incapacity or substantial disruption, congenital anomaly/birth defect, or other important medical events. ADRs were defined as AEs for which a causal relationship with the drug could not be ruled out. Capillary leak syndrome was specified as an AE of special interest. The observation period for AEs was from the time of the first administration of the study drug to 14 days after the last administration of the study drug.

Effectiveness was assessed using the Revised Response Criteria for Malignant Lymphoma [29] for patients with PTCL and using the International Society for Cutaneous Lymphomas/European Organization for Research and Treatment of Cancer Global Response Score criteria [30] for patients with CTCL.

### 2.4 Statistical Methods

Based on the results of the Phase II trial (ClinicalTrials.gov identifier NCT02676778) [20], 85 patients were required to achieve a 90% probability of observing at least one of the least frequently occurring AEs. Therefore, the target sample size was set at 85 patients.

The safety analysis set included all patients who received at least one dose of the treatment. All patients from the safety analysis set who had effectiveness data available were included in the effectiveness analysis set. Analyses were performed in the overall population (PTCL and CTCL combined), as well as separately in patients with PTCL and in patients with CTCL.

The numbers of AEs, grade ≥ 3 AEs and serious AEs, including their incidence rates, were calculated. For ADRs, the number of events, grade ≥ 3 ADRs and serious ADRs, including their incidence rate, were calculated. All were calculated for the overall population in the safety analysis set.

AEs and ADRs were tabulated by Preferred Term using the Medical Dictionary for Regulatory Activities/Japanese version 26.1. Odds ratios (ORs), 95% CIs, and *p*‐values were calculated using univariate logistic regression analysis to examine factors associated with ADRs. Univariate logistic regression analyses were also conducted to examine factors associated with capillary leak syndrome.

For the effectiveness analysis, the best overall response, ORR, disease control rate (DCR), and their 95% CIs were calculated. ORR was defined as the proportion of patients with the best overall response to treatment (complete response [CR] and partial response [PR]), and DCR was defined as the proportion of patients with disease stabilization (CR, PR, and stable disease). ORs, 95% CIs, and *p‐*values were calculated using univariate logistic regression analysis to identify factors associated with response rates.

In both the safety analysis and the effectiveness analysis, if the number of patients/cases was ≤ 1, “–” was the output for both the number of patients/cases and the ratio. Subgroup analyses for ADRs and the effectiveness analysis were conducted by patient background characteristics. All subgroup analyses and univariate logistic regression analyses were exploratory, and no adjustment for multiple testing was performed. The significance level was 5%.

All analyses were performed using SAS version 9.4 (SAS Institute Inc., Cary, NC, USA) and Excel version 2016 (Microsoft Corp., Redmond, WA, USA).

## 3 Results

### 3.1 Patients

A total of 118 patients received DD‐cxdl at the participating institutions in Japan during the registration period, and 111 survey forms were obtained. From these forms, data from 104 patients that could be anonymized were included in the analysis (85 patients with PTCL and 19 with CTCL), including data from 80 patients (65 patients with PTCL and 15 with CTCL) for whom effectiveness evaluations were available. Effectiveness evaluations could not be performed for 24 patients because no such evaluations were conducted in routine clinical practice.

The patient baseline characteristics are shown in Table 1. Approximately half of the patients were male (57/104 [54.8%] overall; 44/85 [51.8%] of patients with PTCL and 13/19 [68.4%] of patients with CTCL). Most patients were aged ≥ 65 years (73/85 [85.9%] patients with PTCL and 14/19 [73.7%] patients with CTCL). For patients with PTCL, 54/85 (63.5%) had PTCL, not otherwise specified; 26/85 (30.6%) had angioimmunoblastic T‐cell lymphoma; and 5/85 (5.9%) had systemic anaplastic large cell lymphoma. For patients with CTCL, 15/19 (78.9%) had mycosis fungoides/Sézary syndrome; and 5/19 (26.3%) had a primary cutaneous CD30^+^ T‐cell lymphoproliferative disorder. For PTCL and CTCL, the numbers (%) of patients who had received ≥ 3 lines of prior chemotherapy were 59/85 (69.4%) and 13/19 (68.4%), respectively. The median (range) number of prior chemotherapy lines was 3.0 (1.0–8.0) in patients with PTCL and 4.0 (1.0–10.0) in patients with CTCL. Thirteen patients with PTCL (15.3%) and two patients with CTCL (10.5%) had received ≥ 6 lines of prior chemotherapy.

**TABLE 1:** Patient baseline characteristics.

	PTCL *n* = 85	CTCL *n* = 19	Overall *N* = 104
Sex			
Female	41 (48.2)	6 (31.6)	47 (45.2)
Male	44 (51.8)	13 (68.4)	57 (54.8)
Age, median (range), years	75 (20–88)	70 (50–78)	74 (20–88)
Age			
< 65 years	12 (14.1)	5 (26.3)	17 (16.3)
≥ 65 years	73 (85.9)	14 (73.7)	87 (83.7)
WHO classification			
PTCL, not otherwise specified	54 (63.5)	—	54 (51.9)
Angioimmunoblastic T‐cell lymphoma	26 (30.6)	—	26 (25.0)
Systemic anaplastic large cell lymphoma	5 (5.9)	—	5 (4.8)
Mycosis fungoides, Sézary syndrome^a^	—	15 (78.9)	15 (14.4)
Primary cutaneous CD30^+^ T‐cell lymphoproliferative disorders^a^	—	5 (26.3)	5 (4.8)
Stage			
II	4 (4.7)	7 (36.8)	11 (10.6)
III	18 (21.2)	3 (15.8)	21 (20.2)
IV	53 (62.4)	9 (47.4)	62 (59.6)
Unknown	10 (11.8)	—	10 (9.6)
Disease status^b^			
Refractory	39 (45.9)	7 (36.8)	46 (44.2)
Relapsed	43 (50.6)	10 (52.6)	53 (51.0)
Unknown	3 (3.5)	2 (10.5)	5 (4.8)
CD25 status			
Positive	23 (27.1)	5 (26.3)	28 (26.9)
Negative	20 (23.5)	4 (21.1)	24 (23.1)
Unknown	42 (49.4)	10 (52.6)	52 (50.0)
CD30 status			
Positive	30 (35.3)	10 (52.6)	40 (38.5)
Negative	37 (43.5)	7 (36.8)	44 (42.3)
Unknown	18 (21.2)	2 (10.5)	20 (19.2)
ECOG‐PS			
0	11 (12.9)	3 (15.8)	14 (13.5)
1	34 (40.0)	8 (42.1)	42 (40.4)
2	22 (25.9)	4 (21.1)	26 (25.0)
3	12 (14.1)	2 (10.5)	14 (13.5)
4	6 (7.1)	2 (10.5)	8 (7.7)
Number of prior chemotherapy lines			
1	10 (11.8)	3 (15.8)	13 (12.5)
2	16 (18.8)	3 (15.8)	19 (18.3)
3	18 (21.2)	2 (10.5)	20 (19.2)
4	14 (16.5)	4 (21.1)	18 (17.3)
5	14 (16.5)	5 (26.3)	19 (18.3)
≥ 6	13 (15.3)	2 (10.5)	15 (14.4)
Median (range)	3.0 (1.0–8.0)	4.0 (1.0–10.0)	3.5 (1.0–10.0)
Combination therapy	12 (14.1)	3 (15.8)	15 (14.4)
Preventive drugs for ADRs	68 (80.0)	14 (73.7)	82 (78.8)
Prior hematopoietic stem cell transplantation	8 (9.4)	—	8 (7.7)
LDH			
< 223 U/L	20 (23.5)	5 (26.3)	25 (24.0)
≥ 223 U/L	63 (74.1)	14 (73.7)	77 (74.0)
Not measured	2 (2.4)	—	2 (1.9)
sIL‐2R			
< 2000 U/mL	17 (20.0)	12 (63.2)	29 (27.9)
≥ 2000 U/mL	51 (60.0)	3 (15.8)	54 (51.9)
Not measured	17 (20.0)	4 (21.1)	21 (20.2)

*Note:* Data shown as *n* (%), unless otherwise stated.

Abbreviations: ADR, adverse drug reaction; CTCL, cutaneous T‐cell lymphoma; ECOG‐PS, Eastern Cooperative Oncology Group Performance Status; LDH, lactate dehydrogenase; PTCL, peripheral T‐cell lymphoma; sIL‐2R, soluble interleukin‐2 receptor; WHO, World Health Organization.

^a^ Including patients with overlapping diagnoses.

^b^ Refractory disease was defined as a tumor evaluation of progressive disease after the most recent chemotherapy. Relapsed disease was defined as a tumor evaluation of complete response, partial response, or stable disease after the most recent chemotherapy. If no information on the most recent chemotherapy or tumor evaluation was available, or if the tumor evaluation was not evaluated or unknown, the patient's disease status was categorized as unknown.

### 3.2 Treatment

The initial dose received by patients and the number of cycles are shown in Table 2. Most patients with PTCL and those with CTCL received an initial dose of 9.0 μg/kg (67/85 [78.8%] and 16/19 [84.2%], respectively). For both patients with PTCL and those with CTCL, the median number of cycles was 1.0.

**TABLE 2:** Denileukin diftitox‐cxdl treatment.

	PTCL *n* = 85	CTCL *n* = 19	Overall *N* = 104
Initial dose, median (range), μg/kg	9.0 (4.9–9.0)	9.0 (4.5–9.0)	9.0 (4.5–9.0)
Initial dose category			
< 9.0 μg/kg	18 (21.2)	3 (15.8)	21 (20.2)
9.0 μg/kg	67 (78.8)	16 (84.2)	83 (79.8)
Final cycle^a^			
1	48 (56.5)	10 (52.6)	58 (55.8)
2	16 (18.8)	5 (26.3)	21 (20.2)
3	5 (5.9)	2 (10.5)	7 (6.7)
≥ 4	16 (18.8)	2 (10.5)	18 (17.3)
Median number of cycles	1.0	1.0	1.0
Treatment discontinuations	81 (95.3)	18 (94.7)	99 (95.2)
Reason for treatment discontinuation			
Disease progression	52 (61.2)	10 (52.6)	62 (59.6)
Adverse event	38 (44.7)	7 (36.8)	45 (43.3)
Patient's decision	7 (8.2)	—	8 (7.7)
Other	9 (10.6)	—	10 (9.6)

*Note:* Data shown as *n* (%) or *n*, unless otherwise stated.

Abbreviations: CTCL, cutaneous T‐cell lymphoma; PTCL, peripheral T‐cell lymphoma.

^a^ The final cycle was the cycle during which the patient discontinued treatment.

In the overall population, 58/104 (55.8%) patients discontinued treatment during cycle 1, 21/104 (20.2%) during cycle 2, 7/104 (6.7%) during cycle 3, and 18/104 (17.3%) during or after cycle 4. Among patients with PTCL, 48/85 (56.5%) patients discontinued treatment during cycle 1, 16/85 (18.8%) during cycle 2, 5/85 (5.9%) during cycle 3, and 16/85 (18.8%) during or after cycle 4. Among those with CTCL, 10/19 (52.6%) patients discontinued treatment during cycle 1, 5/19 (26.3%) during cycle 2, 2/19 (10.5%) during cycle 3, and 2/19 (10.5%) during or after cycle 4.

Most of the patients who discontinued treatment (Table S1) during cycle 1 were ≥ 65 years of age (48/58 [82.8%]). The median (range) number of prior chemotherapy lines was 4.0 (1.0–10.0); most of these patients had received ≥ 3 prior lines of chemotherapy (42/58 [72.4%]), while 8/58 (13.8%) had received ≥ 6 prior lines of chemotherapy. Compared with the overall population, patients who discontinued treatment during cycle 1 had a numerically higher percentage of patients with Stage IV disease (59.6% vs. 67.2%), ECOG‐PS scores ≥ 2 (46.2% vs. 53.4%), lactate dehydrogenase (LDH) ≥ 223 U/L (74.0% vs. 79.3%), and soluble interleukin‐2 receptor (sIL‐2R) ≥ 2000 U/mL (51.9% vs. 56.9%).

The most common reason for discontinuation overall and among patients who discontinued treatment during cycle 1 was disease progression (62/104 [59.6%] and 36/58 [62.1%], respectively). The most common AEs that led to discontinuation among patients who discontinued treatment during cycle 1 were capillary leak syndrome and hepatic function abnormal (each 5/58 [8.6%]).

### 3.3 Safety

The overall incidence of AEs was 98/104 (94.2%), and the overall incidence of ADRs was 88/104 (84.6%). Grade ≥ 3 AEs occurred in 65/104 (62.5%) patients and grade ≥ 3 ADRs occurred in 55/104 (52.9%). Overall, 56/104 (53.8%) patients experienced serious AEs and 43/104 (41.3%) experienced serious ADRs. The most common ADRs (Table 3) were aspartate aminotransferase increased and capillary leak syndrome (both 23/104 cases [22.1%]), alanine aminotransferase increased (22/104 cases [21.2%]), hepatic function abnormal (16/104 cases [15.4%]), platelet count decreased (15/104 cases [14.4%]), and pyrexia (13/104 cases [12.5%]). Among grade ≥ 3 ADRs, the most common was platelet count decreased (11/104 cases [10.6%]), followed by capillary leak syndrome and hepatic function abnormal (both 9/104 cases [8.7%]). The most common serious ADR was capillary leak syndrome (23/104 cases [22.1%]), followed by pyrexia (4/104 cases [3.8%]). All 23 capillary leak syndrome cases were serious ADRs; 7/23 (30.4%) resulted in treatment discontinuations, and 4/23 (17.4%) in delayed drug administration.

**TABLE 3:** Most common and major adverse drug reactions in the overall population.

ADRs (occurrence in ≥ 2 patients)	Any grade *N* = 104	Grade ≥ 3 *N* = 104
Overall	88 (84.6)	55 (52.9)
Aspartate aminotransferase increased	23 (22.1)	8 (7.7)
Capillary leak syndrome	23 (22.1)	9 (8.7)
Alanine aminotransferase increased	22 (21.2)	8 (7.7)
Hepatic function abnormal	16 (15.4)	9 (8.7)
Platelet count decreased	15 (14.4)	11 (10.6)
Pyrexia	13 (12.5)	2 (1.9)
Rash	7 (6.7)	—
Decreased appetite	7 (6.7)	—
Hypoalbuminemia	6 (5.8)	—
Lymphocyte count decreased	6 (5.8)	4 (3.8)
Febrile neutropenia	5 (4.8)	4 (3.8)
Liver disorder	5 (4.8)	—
Anemia	4 (3.8)	—
Malaise	4 (3.8)	—
Nausea	4 (3.8)	—
Blood albumin decreased	3 (2.9)	—
Cytomegalovirus infection	3 (2.9)	3 (2.9)
Erythema	3 (2.9)	2 (1.9)
Pneumonia	3 (2.9)	—
Skin infection	3 (2.9)	3 (2.9)
Infusion related reaction	3 (2.9)	2 (1.9)
Cytomegalovirus viremia	3 (2.9)	—
Hepatic enzyme increased	3 (2.9)	—
Atrial fibrillation	2 (1.9)	—
Blood creatine phosphokinase increased	2 (1.9)	—
Blood lactate dehydrogenase increased	2 (1.9)	—
Infection	2 (1.9)	—
Sepsis	2 (1.9)	—
Thrombocytopenia	2 (1.9)	2 (1.9)
Vomiting	2 (1.9)	—

*Note:* Data shown as *n* (%).

Abbreviation: ADR, adverse drug reaction.

No patient background characteristics were identified as having a statistically significant relationship with the incidence of ADRs (Table S2). The incidence rate of capillary leak syndrome by patient characteristics is shown in Table S3. ORs were 0.332 (95% CI: 0.106, 0.975; *p* = 0.045) for age ≥ 65 years, 0.199 (0.054, 0.735; *p* = 0.015) for disease Stage III or IV, 0.328 (0.117, 0.916; *p* = 0.033) for ECOG‐PS scores ≥ 2, and 0.212 (0.077, 0.586; *p* = 0.003) for LDH ≥ 223 U/L.

### 3.4 Effectiveness

The summary of effectiveness evaluations is shown in Table 4. Overall, the ORR was 16.3% (95% CI: 8.9, 26.2). The ORR was 15.4% (95% CI: 7.6, 26.5) in patients with PTCL and 20.0% (95% CI: 4.3, 48.1) in patients with CTCL. Four patients achieved CR, all of whom had PTCL. The DCR was 48.8% (95% CI: 37.4, 60.2), 46.2% (95% CI: 33.7, 59.0), and 60.0% (95% CI: 32.3, 83.7) overall, in patients with PTCL, and in patients with CTCL, respectively.

**TABLE 4:** Response rate and disease control rate.

	PTCL *n* = 65	CTCL *n* = 15	Overall *N* = 80
Best overall response, *n* (%)			
Complete response	4 (6.2)	0 (0.0)	4 (5.0)
Partial response	6 (9.2)	3 (20.0)	9 (11.3)
Stable disease	20 (30.8)	6 (40.0)	26 (32.5)
Progressive disease	35 (53.8)	6 (40.0)	41 (51.3)
Objective response rate [95% CI]	15.4% [7.6, 26.5]	20.0% [4.3, 48.1]	16.3% [8.9, 26.2]
Disease control rate [95% CI]	46.2% [33.7, 59.0]	60.0% [32.3, 83.7]	48.8% [37.4, 60.2]

Abbreviations: CI, confidence interval; CTCL, cutaneous T‐cell lymphoma; PTCL, peripheral T‐cell lymphoma.

The factors associated with ORR and DCR are shown in Table 5. In the exploratory univariate analysis, relapsed disease was associated with a higher DCR (OR: 2.738 [95% CI: 1.088, 6.888], *p* = 0.032), whereas no factor was significantly associated with ORR.

**TABLE 5:** Response rate and disease control rate by patient characteristics.

	*n*	Patients with CR or PR	Odds ratio [95% CI]	*p*	Patients with CR, PR, or SD	Odds ratio [95% CI]	*p*
Overall	80	13 (16.3)			39 (48.8)		
Sex							
Female	35	4 (11.4)	0.516 [0.145, 1.841]	0.308	14 (40.0)	0.533 [0.218, 1.307]	0.169
Male	45	9 (20.0)	Reference		25 (55.6)	Reference	
Age							
< 65 years	15	4 (26.7)	Reference		9 (60.0)	Reference	
≥ 65 years	65	9 (13.8)	0.442 [0.115, 1.694]	0.234	30 (46.2)	0.571 [0.182, 1.791]	0.337
Age category							
< 30 years	2	—			2 (100.0)		
30 to < 45 years	3	—			—		
45 to < 60 years	4	—			3 (75.0)		
60 to < 75 years	33	7 (21.2)			15 (45.5)		
≥ 75 years	38	4 (10.5)			18 (47.4)		
WHO classification							
PTCL, not otherwise specified	40	6 (15.0)			20 (50.0)		
Angioimmunoblastic T‐cell lymphoma	20	4 (20.0)			9 (45.0)		
Systemic anaplastic large cell lymphoma	5	—			—		
Mycosis fungoides, Sézary syndrome^a^	12	3 (25.0)			9 (75.0)		
Primary cutaneous CD30^+^ T‐cell lymphoproliferative disorders^a^	4	—			—		
Stage							
II	9	2 (22.2)			6 (66.7)		
III	18	4 (22.2)			9 (50.0)		
IV	47	6 (12.8)			22 (46.8)		
Unknown	6	—			2 (33.3)		
Stages							
I, II	9	2 (22.2)	Reference		6 (66.7)	Reference	
III, IV	65	10 (15.4)	0.636 [0.115, 3.518]	0.604	31 (47.7)	0.456 [0.105, 1.981]	0.295
Unknown	6	—			2 (33.3)		
Disease status^b^							
Refractory	37	6 (16.2)	Reference		14 (37.8)	Reference	
Relapsed	40	7 (17.5)	1.096 [0.332, 3.623]	0.881	25 (62.5)	2.738 [1.088, 6.888]	0.032
Unknown	3	—			—		
CD25 status							
Positive	22	5 (22.7)	Reference		13 (59.1)	Reference	
Negative	16	2 (12.5)	0.486 [0.081, 2.897]	0.428	7 (43.8)	0.538 [0.146, 1.982]	0.352
Unknown	42	6 (14.3)			19 (45.2)		
CD30 status							
Positive	30	5 (16.7)	Reference		17 (56.7)	Reference	
Negative	31	5 (16.1)	0.962 [0.248, 3.730]	0.955	13 (41.9)	0.552 [0.200, 1.524]	0.252
Unknown	19	3 (15.8)			9 (47.4)		
ECOG‐PS							
0	13	3 (23.1)			10 (76.9)		
1	30	6 (20.0)			13 (43.3)		
2	21	—			7 (33.3)		
3	12	2 (16.7)			7 (58.3)		
4	4	—			2 (50.0)		
ECOG‐PS							
0 or 1	43	9 (20.9)	Reference		23 (53.5)	Reference	
≥ 2	37	4 (10.8)	0.458 [0.128, 1.633]	0.229	16 (43.2)	0.663 [0.274, 1.604]	0.362
Number of prior chemotherapy lines							
1	11	2 (18.2)			3 (27.3)		
2	11	—			5 (45.5)		
3	15	3 (20.0)			9 (60.0)		
4	16	3 (18.8)			9 (56.3)		
5	15	2 (13.3)			6 (40.0)		
≥ 6	12	3 (25.0)			7 (58.3)		
Number of prior chemotherapy lines							
≤ 2	22	2 (9.1)	Reference		8 (36.4)	Reference	
≥ 3	58	11 (19.0)	2.340 [0.475, 11.533]	0.296	31 (53.4)	2.009 [0.732, 5.518]	0.176
Combination therapy							
No	68	11 (16.2)	Reference		33 (48.5)	Reference	
Yes	12	2 (16.7)	1.037 [0.199, 5.396]	0.966	6 (50.0)	1.061 [0.311, 3.619]	0.925
Preventive drugs for ADRs							
No	16	2 (12.5)	Reference		7 (43.8)	Reference	
Yes	64	11 (17.2)	1.452 [0.288, 7.320]	0.651	32 (50.0)	1.286 [0.427, 3.873]	0.655
Prior hematopoietic stem cell transplantation							
No	72	10 (13.9)	Reference		33 (45.8)	Reference	
Yes	8	3 (37.5)	3.720 [0.767, 18.052]	0.103	6 (75.0)	3.545 [0.670, 18.761]	0.137
LDH							
< 223 U/L	20	5 (25.0)	Reference		12 (60.0)	Reference	
≥ 223 U/L	58	7 (12.1)	0.412 [0.114, 1.487]	0.176	26 (44.8)	0.542 [0.193, 1.523]	0.245
Not measured	2	—			—		
sIL‐2R							
< 2000 U/mL	24	6 (25.0)	Reference		13 (54.2)	Reference	
≥ 2000 U/mL	41	3 (7.3)	0.237 [0.053, 1.056]	0.059	18 (43.9)	0.662 [0.241, 1.823]	0.425
Not measured	15	4 (26.7)			8 (53.3)		

*Note:* Data shown as *n* or *n* (%), unless otherwise stated.

Abbreviations: ADR, adverse drug reaction; CI, confidence interval; CR, complete response; ECOG‐PS, Eastern Cooperative Oncology Group Performance Status; LDH, lactate dehydrogenase; PR, partial response; SD, stable disease; sIL‐2R, soluble interleukin‐2 receptor; WHO, World Health Organization.

^a^ Including patients with overlapping diagnoses.

^b^ Refractory disease was defined as a tumor evaluation of progressive disease after the most recent chemotherapy. Relapsed disease was defined as a tumor evaluation of complete response, partial response, or stable disease after the most recent chemotherapy. If no information on the most recent chemotherapy or tumor evaluation was available, or if the tumor evaluation was not evaluated or unknown, the patient's disease status was categorized as unknown.

## 4 Discussion

This all‐case post‐marketing study provides the first real‐world insights on the safety and effectiveness of DD‐cxdl in daily clinical practice for patients with relapsed or refractory PTCL and CTCL in Japan. Most patients experienced some form of AEs (94.2%) or ADRs (84.6%), with capillary leak syndrome (22.1%) as the most common serious ADR. Despite the heavily pretreated nature of the cohort, antitumor activity was observed overall (ORR in the overall population was 16.3% [95% CI: 8.9, 26.2]), and in both the PTCL and CTCL populations (ORR in patients with PTCL and CTCL: 15.4% [95% CI: 7.6, 26.5] and 20.0% [95% CI: 4.3, 48.1], respectively), including a subset of patients (*n* = 4; 6.2%) with PTCL who achieved CR. Moreover, the DCR in the overall population was 48.8% (95% CI: 37.4, 60.2), and the DCR in patients with PTCL and CTCL was 46.2% (95% CI: 33.7, 59.0) and 60.0% (95% CI: 32.3, 83.7), respectively.

In the previous Phase I and II trials of E7777, patients received a median of three [22] and four [20] DD‐cxdl treatment cycles, respectively. In contrast, patients in this post‐marketing study received a median of one treatment cycle. Additionally, the ORR achieved in the current study was lower than reported in the previous trials [20, 22]. It is possible that patients in this study did not receive DD‐cxdl treatment for a sufficiently long duration. Although many patients experienced treatment interruption during cycles 1 and 2 in the Phase III trial, patients were able to continue after resuming treatment at the full dose. This resulted in a median of six DD‐cxdl treatment cycles and an ORR of 36.2% [23]. Thus, we believe it is important to ensure an adequate treatment duration, beyond the first cycle, to improve the ORR.

Compared with the Phase I and II trials [20, 22], this study included patients with a wider range of ages, ECOG‐PS scores, and a larger number of prior chemotherapy lines. The median (range) age of patients in the Phase I trial was 58 (23–75) years and 65 (27–82) years in the Phase II trial [20, 22], while the median age of the patients included in this study was 74 (20–88) years. The inclusion criteria of the previous clinical trials required patients to have an ECOG‐PS score of 0 or 1 [20, 22], while patients in this study had scores ranging from 0 to 4. Approximately half of the patients in the Phase I and Phase II trials had received < 2 prior chemotherapy lines [20, 22], whereas almost 70% of the patients in this study had received ≥ 3 prior chemotherapy lines. Thus, more heavily pretreated older patients with a worse performance status were included in this study than in earlier trials [20, 22]. In the exploratory analysis of this study, relapsed disease was associated with a higher DCR. Additionally, patients who received ≥ 3 prior chemotherapy lines showed a numerically higher DCR. However, these findings should be interpreted cautiously because they were derived from the exploratory subgroup analyses without adjustment for multiple testing and may be influenced by patient selection, early discontinuation, and small subgroup sizes. In the Phase III trial in patients with CTCL, the ORR was 36.2% (95% CI: 25.0, 48.7) [23], which is higher than observed in the present study. In this study, more than half of patients had Stage IV disease, whereas all patients in the Phase III trial had Stage I–III disease. Thus, the difference in ORR may be attributable to the differences in disease stage. The DCR in patients with CTCL in this study was numerically higher than in the Phase III trial (DCR: 49.3%, 95% CI: 37.0, 61.6) [23]. This comparison should also be interpreted cautiously, as the Phase III trial included only patients with CTCL, while the present study included both patients with PTCL and CTCL and reflects daily clinical practice. Thus, we postulate that the higher DCR in patients with CTCL observed in the present study may be attributed to our methodology. This study used best overall response for evaluation, whereas the Phase III trial relied on durable response evaluation, which only reflected achievement of stable disease if this condition was maintained throughout the first 23 weeks of DD‐cxdl administration [23]. Although the ORR was lower due to the short DD‐cxdl treatment cycle, our results suggest that disease control is achievable.

Two Phase III trials of DD treatment in patients with CD25‐positive (> 20% of tumor cells with CD25) CTCL achieved an ORR of 30% and 44% [16, 17]. However, other clinical trials of DD‐cxdl in CTCL patients have reported that ORR is not dependent on CD25 expression [31]. In addition, the Phase III trial of DD‐cxdl demonstrated no correlation between CD25‐positivity and efficacy. Therefore, the Food and Drug Administration did not require a companion diagnostic test for CD25. Similarly, in this post‐marketing study, no statistically significant difference was observed between CD25 expression levels and ORR.

None of the background factors evaluated were associated with the occurrence of ADRs, and the frequency of serious AEs in this study was consistent with the previous Phase II trial [20]. In the Phase II trial, the incidence of capillary leak syndrome was 13.5% for any grade and 10.8% for grade ≥ 3. In this study, the incidence of capillary leak syndrome was 22.1% for any grade and 8.7% for grade ≥ 3. The incidence rate of capillary leak syndrome of any grade in this study was higher than that in the Phase II trial, but this may be attributable to differences in diagnostic criteria between daily clinical practice and clinical trials. In clinical trials, clear criteria for capillary leak syndrome (requiring two or more defined symptoms) were defined, but in daily clinical practice, diagnosis may have been established using more lenient criteria. Although some factors were associated with lower odds of developing capillary leak syndrome, these findings should be interpreted cautiously as they may reflect confounding inherent to post‐marketing surveillance, such as early discontinuation, competing risks, under‐reporting, or the small sample size. The lower incidence of grade ≥ 3 capillary leak syndrome in this study may reflect differences in recognition, diagnostic thresholds, and clinical management in daily practice; however, data on specific management strategies and outcomes were not collected, precluding firm conclusions.

We acknowledge the limitations of this study. This study was based on case report forms completed by each physician, and the source data verification used in clinical trials was not conducted. Additionally, some detailed information was not collected. Effectiveness evaluations were performed at the physician's discretion, and these were not reviewed by an independent committee. Additionally, the timing of the evaluations was not defined in detail as this was a retrospective study. Moreover, it is unclear whether the effectiveness criteria [29, 30] were applied consistently across all institutions because the details of the assessment procedures at each participating institution are unknown, and the assessment of skin conditions using the Modified Severity Weighted Assessment Tool is challenging. Not all patients had available effectiveness evaluation data, and many discontinued DD‐cxdl treatment and received short cycles. Because PTCL and CTCL are rare diseases, some subtypes were not well represented. Because all patients were from Japan, there are limitations regarding the generalizability of the findings to patients from different geographic regions. Multiple subgroup comparisons were exploratory and conducted without adjustment for multiple testing, with some subgroups only including a small number of patients. Thus, the results of these analyses should be interpreted cautiously and considered hypothesis‐generating rather than confirmatory. Despite these limitations, the strengths of this study are its relatively large sample size (including the largest sample size of patients with PTCL to date) and the variety of patients included, which provide confidence that the findings are likely to reflect daily clinical practice.

In conclusion, this is the first observational study to evaluate the safety and effectiveness of DD‐cxdl in patients with relapsed or refractory PTCL and CTCL in Japanese daily clinical practice. The observed safety profile of DD‐cxdl was generally consistent with the previously reported profile in Phase I and II trials. No new safety concerns were identified; however, clinicians should remain vigilant for the risk of capillary leak syndrome. DD‐cxdl may be feasible in later lines of therapy, but it could be more effective when used in earlier settings, allowing for adequate treatment duration. Given the limited availability of systemic treatments, DD‐cxdl represents a potential therapeutic option for patients with relapsed/refractory PTCL and CTCL.

## Author Contributions

**Kota Matsumoto:** conceptualization, methodology, investigation, project administration, resources, validation, visualization, writing – original draft, writing – review and editing. **Tatsuro Jo:** conceptualization, funding acquisition, investigation, supervision, visualization, writing – original draft, writing – review and editing. **Mika Ishii:** conceptualization, investigation, methodology, project administration, resources, visualization, writing – original draft, writing – review and editing. **Koji Izutsu:** writing – original draft, writing – review and editing, visualization, supervision, resources, methodology, investigation, conceptualization.

## Funding

This study was funded by Eisai Co. Ltd. Eisai Co. Ltd. was involved in the study design, data collection, analysis, interpretation, manuscript preparation, and the decision to submit the manuscript for publication. Other than Eisai Co. Ltd. as described above, the companies disclosed in the Conflict of Interest Statement had no involvement in this study or this publication.

## Ethics Statement

This study was conducted according to the Japanese Good Post‐Marketing Study Practice (GPSP; the Japanese Ministry of Health, Labour and Welfare Ordinance No. 171, 2004). According to the Guidance for the Japanese Ethical Guidelines for Medical and Biological Research Involving Human Subjects, a post‐marketing study conducted in accordance with the GPSP is outside the scope of these Ethical Guidelines. Therefore, informed consent and institutional review board approval under these Ethical Guidelines were not required. Nevertheless, this study was centrally approved by the Clinical Research Advisory Board of Eisai Co. Ltd. on December 17, 2019. Patient data were anonymized to ensure confidentiality and privacy.

This study was registered on ClinicalTrials.gov under the identifier NCT05137847 and on the Japan Registry of Clinical Trials under the identifier jRCT2031220196.

## Conflicts of Interest

Tatsuro Jo received support for medical writing, provision of study materials, article processing charges, and advisory fees for the present manuscript from Eisai Co. Ltd.; and payment or honoraria for lectures, presentations, speakers' bureaus, and manuscript writing or educational events from Eisai Co. Ltd., and Bristol‐Myers Squibb K.K. Kota Matsumoto and Mika Ishii are employees of Eisai Co. Ltd. Koji Izutsu received research funding for the present manuscript from Eisai Co. Ltd.; and research funding from AstraZeneca K.K., AbbVie G.K., BeiGene Ltd., Bristol‐Myers Squibb K.K., Chugai Pharmaceutical Co. Ltd., Daiichi Sankyo Co. Ltd., Genmab K.K. Incyte Biosciences Japan G.K., Loxo Oncology Inc., Novartis Pharma K.K., Otsuka Pharmaceutical Co. Ltd., Yakult Honsha Co. Ltd., and Regeneron Japan K.K.

## Supporting information


**Table S1:** Characteristics of patients who discontinued treatment during cycle 1.
**Table S2:** Incidence rate of ADRs by patient characteristics.
**Table S3:** Incidence rate of capillary leak syndrome by patient characteristics.

## Data Availability

The data that support the findings of this study are available on request from the corresponding author. The data are not publicly available due to privacy or ethical restrictions.
