# Supplementary material for: Safety and Effectiveness of Denileukin Diftitox‐cxdl in Patients With Relapsed/Refractory Peripheral T‐Cell Lymphoma and Cutaneous T‐Cell Lymphoma: An All‐Case Post‐Marketing Study in Japan
Source: Cancer Rep (Hoboken). 2026 Aug 16;9(8):e70641. doi: 10.1002/cnr2.70641 (PMC13478371; doi:10.1002/cnr2.70641)
Supplement: Supplementary file 1 [file CNR2-9-e70641-s001.docx]

**Table S1.** Characteristics of patients who discontinued treatment during cycle 1.

|  | ***N* = 58** |
| --- | --- |
| Sex |  |
| Female | 25 (43.1) |
| Male | 33 (56.9) |
| Age, median (range), years | 74 (20–88) |
| Age |  |
| <65 years | 10 (17.2) |
| ≥65 years | 48 (82.8) |
| WHO classification |  |
| PTCL, not otherwise specified | 31 (53.4) |
| Angioimmunoblastic T-cell lymphoma | 13 (22.4) |
| Systemic anaplastic large cell lymphoma | 4 (6.9) |
| Mycosis fungoides, Sézary syndrome† | 9 (15.5) |
| Stage |  |
| II | 5 (8.6) |
| III | 10 (17.2) |
| IV | 39 (67.2) |
| Unknown | 4 (6.9) |
| Disease status‡ |  |
| Refractory | 25 (43.1) |
| Relapsed | 31 (53.4) |
| Unknown | 2 (3.4) |
| CD25 status |  |
| Positive | 18 (31.0) |
| Negative | 13 (22.4) |
| Unknown | 27 (46.6) |
| CD30 status |  |
| Positive | 21 (36.2) |
| Negative | 29 (50.0) |
| Unknown | 8 (13.8) |
| ECOG-PS |  |
| 0 | 6 (10.3) |
| 1 | 21 (36.2) |
| 2 | 18 (31.0) |
| 3 | 8 (13.8) |
| 4 | 5 (8.6) |
| Number of prior chemotherapy lines |  |
| 1 | 4 (6.9) |
| 2 | 12 (20.7) |
| 3 | 10 (17.2) |
| 4 | 11 (19.0) |
| 5 | 13 (22.4) |
| ≥6 | 8 (13.8) |
| Median (range) | 4.0 (1.0–10.0) |
| Combination therapy | 6 (10.3) |
| Preventive drugs for ADRs | 47 (81.0) |
| Prior hematopoietic stem cell transplantation | 4 (6.9) |
| LDH |  |
| <223 U/L | 12 (20.7) |
| ≥223 U/L | 46 (79.3) |
| sIL-2R |  |
| <2000 U/mL | 14 (24.1) |
| ≥2000 U/mL | 33 (56.9) |
| Not measured | 11 (19.0) |

Data shown as *n* (%), unless otherwise stated.

†Including patients with overlapping diagnoses.

‡Refractory disease was defined as a tumor evaluation of progressive disease after the most recent chemotherapy. Relapsed disease was defined as a tumor evaluation of complete response, partial response, or stable disease after the most recent chemotherapy. If no information on the most recent chemotherapy or tumor evaluation was available, or if the tumor evaluation was not evaluated or unknown, the patient’s disease status was categorized as unknown.

ADR, adverse drug reaction; ECOG-PS, Eastern Cooperative Oncology Group Performance Status; LDH, lactate dehydrogenase; PTCL, peripheral T-cell lymphoma; sIL-2R, soluble interleukin-2 receptor; WHO, World Health Organization.

**Table S2.** Incidence rate of ADRs by patient characteristics

|  | Overall | | | | | |
| --- | --- | --- | --- | --- | --- | --- |
|  | *N* | Number of patients with ADRs | Proportion of patients with ADRs (%) | Odds ratio | 95% CI | *P* value |
| Overall | 104 | 88 | 84.6 |  |  |  |
| Sex |  |  |  |  |  |  |
| Male | 57 | 48 | 84.2 | Reference |  |  |
| Female | 47 | 40 | 85.1 | 1.071 | 0.366, 3.134 | 0.900 |
| Age |  |  |  |  |  |  |
| <65 years | 17 | 15 | 88.2 | Reference |  |  |
| ≥65 years | 87 | 73 | 83.9 | 0.696 | 0.143, 3.384 | 0.653 |
| Age category |  |  |  |  |  |  |
| <30 years | 2 | 2 | 100.0 |  |  |  |
| 30 to <45 years | 3 | 2 | 66.7 |  |  |  |
| 45 to <60 years | 4 | 3 | 75.0 |  |  |  |
| 60 to <75 years | 48 | 42 | 87.5 |  |  |  |
| ≥75 years | 47 | 39 | 83.0 |  |  |  |
| WHO classification |  |  |  |  |  |  |
| PTCL, not otherwise specified | 54 | 43 | 79.6 |  |  |  |
| Angioimmunoblastic T-cell lymphoma | 26 | 23 | 88.5 |  |  |  |
| Systemic anaplastic large cell lymphoma | 5 | 4 | 80.0 |  |  |  |
| Mycosis fungoides, Sézary syndrome† | 15 | 14 | 93.3 |  |  |  |
| Primary cutaneous CD30^+^ T-cell lymphoproliferative disorders† | 5 | 5 | 100.0 |  |  |  |
| Stage |  |  |  |  |  |  |
| II | 11 | 11 | 100.0 |  |  |  |
| III | 21 | 18 | 85.7 |  |  |  |
| IV | 62 | 50 | 80.6 |  |  |  |
| Unknown | 10 | 9 | 90.0 |  |  |  |
| Stages |  |  |  |  |  |  |
| I, II | 11 | 11 | 100.0 | Reference |  |  |
| III, IV | 83 | 68 | 81.9 | N/A | N/A | 0.969 |
| Unknown | 10 | 9 | 90.0 |  |  |  |
| Disease status‡ |  |  |  |  |  |  |
| Refractory | 46 | 39 | 84.8 | Reference |  |  |
| Relapsed | 53 | 44 | 83.0 | 0.877 | 0.299, 2.578 | 0.812 |
| Unknown | 5 | 5 | 100.0 |  |  |  |
| CD25 status |  |  |  |  |  |  |
| Positive | 28 | 24 | 85.7 | Reference |  |  |
| Negative | 24 | 17 | 70.8 | 0.405 | 0.102, 1.604 | 0.198 |
| Unknown | 52 | 47 | 90.4 |  |  |  |
| CD30 status |  |  |  |  |  |  |
| Positive | 40 | 37 | 92.5 | Reference |  |  |
| Negative | 44 | 34 | 77.3 | 0.276 | 0.070, 1.087 | 0.066 |
| Unknown | 20 | 17 | 85.0 |  |  |  |
| ECOG-PS |  |  |  |  |  |  |
| 0 | 14 | 12 | 85.7 |  |  |  |
| 1 | 42 | 37 | 88.1 |  |  |  |
| 2 | 26 | 22 | 84.6 |  |  |  |
| 3 | 14 | 12 | 85.7 |  |  |  |
| 4 | 8 | 5 | 62.5 |  |  |  |
| ECOG-PS |  |  |  |  |  |  |
| 0 or 1 | 56 | 49 | 87.5 | Reference |  |  |
| ≥2 | 48 | 39 | 81.3 | 0.619 | 0.212, 1.811 | 0.381 |
| Number of prior chemotherapy lines |  |  |  |  |  |  |
| 1 | 13 | 11 | 84.6 |  |  |  |
| 2 | 19 | 13 | 68.4 |  |  |  |
| 3 | 20 | 19 | 95.0 |  |  |  |
| 4 | 18 | 13 | 72.2 |  |  |  |
| 5 | 19 | 18 | 94.7 |  |  |  |
| ≥6 | 15 | 14 | 93.3 |  |  |  |
| Number of prior chemotherapy lines |  |  |  |  |  |  |
| ≤2 | 32 | 24 | 75.0 | Reference |  |  |
| ≥3 | 72 | 64 | 88.9 | 2.667 | 0.900, 7.904 | 0.077 |
| Combination Therapy |  |  |  |  |  |  |
| No | 89 | 75 | 84.3 | Reference |  |  |
| Yes | 15 | 13 | 86.7 | 1.213 | 0.246, 5.975 | 0.812 |
| Preventive drugs for ADRs |  |  |  |  |  |  |
| No | 22 | 18 | 81.8 | Reference |  |  |
| Yes | 82 | 70 | 85.4 | 1.296 | 0.373, 4.500 | 0.683 |
| Prior hematopoietic stem cell transplantation |  |  |  |  |  |  |
| No | 96 | 80 | 83.3 | Reference |  |  |
| Yes | 8 | 8 | 100.0 | N/A | N/A | 0.974 |
| LDH |  |  |  |  |  |  |
| <223 U/L | 25 | 22 | 88.0 | Reference |  |  |
| ≥223 U/L | 77 | 64 | 83.1 | 0.672 | 0.175, 2.579 | 0.562 |
| Not measured | 2 | 2 | 100.0 |  |  |  |
| sIL-2R |  |  |  |  |  |  |
| <2000 U/mL | 29 | 26 | 89.7 | Reference |  |  |
| ≥2000 U/mL | 54 | 43 | 79.6 | 0.451 | 0.115, 1.768 | 0.253 |
| Not measured | 21 | 19 | 90.5 |  |  |  |

†Including patients with overlapping diagnoses.

‡Refractory disease was defined as a tumor evaluation of progressive disease after the most recent chemotherapy. Relapsed disease was defined as a tumor evaluation of complete response, partial response, or stable disease after the most recent chemotherapy. If no information on the most recent chemotherapy or tumor evaluation was available, or if the tumor evaluation was not evaluated or unknown, the patient’s disease status was categorized as unknown.

ADR, adverse drug reaction; CI, confidence interval; ECOG-PS, Eastern Cooperative Oncology Group Performance Status; LDH, lactate dehydrogenase; N/A, not available; PTCL, peripheral T-cell lymphoma; sIL-2R, soluble interleukin-2 receptor; WHO, World Health Organization.

**Table S3.** Incidence rate of capillary leak syndrome by patient characteristics

|  | Overall | | | | | |
| --- | --- | --- | --- | --- | --- | --- |
|  | *N* | Number of patients with ADRs | Proportion of patients with ADRs (%) | Odds ratio | 95% CI | *P* value |
| Overall | 104 | 23 | 22.1 |  |  |  |
| Sex |  |  |  |  |  |  |
| Male | 57 | 14 | 24.6 | Reference |  |  |
| Female | 47 | 9 | 19.1 | 0.727 | 0.283, 1.870 | 0.509 |
| Age |  |  |  |  |  |  |
| <65 years | 17 | 7 | 41.2 | Reference |  |  |
| ≥65 years | 87 | 16 | 18.4 | 0.322 | 0.106, 0.975 | 0.045 |
| Age category |  |  |  |  |  |  |
| <30 years | 2 | - | - |  |  |  |
| 30 to <45 years | 3 | - | - |  |  |  |
| 45 to <60 years | 4 | 3 | 75.0 |  |  |  |
| 60 to <75 years | 48 | 5 | 10.4 |  |  |  |
| ≥75 years | 47 | 13 | 27.7 |  |  |  |
| WHO classification |  |  |  |  |  |  |
| PTCL, not otherwise specified | 54 | 11 | 20.4 |  |  |  |
| Angioimmunoblastic T-cell lymphoma | 26 | 5 | 19.2 |  |  |  |
| Systemic anaplastic large cell lymphoma | 5 | - | - |  |  |  |
| Mycosis fungoides, Sézary syndrome† | 15 | 7 | 46.7 |  |  |  |
| Primary cutaneous CD30^+^ T-cell lymphoproliferative disorders† | 5 | - | - |  |  |  |
| Stage |  |  |  |  |  |  |
| II | 11 | 6 | 54.5 |  |  |  |
| III | 21 | 6 | 28.6 |  |  |  |
| IV | 62 | 10 | 16.1 |  |  |  |
| Unknown | 10 | - | - |  |  |  |
| Stages |  |  |  |  |  |  |
| I, II | 11 | 6 | 54.5 | Reference |  |  |
| III, IV | 83 | 16 | 19.3 | 0.199 | 0.054, 0.735 | 0.015 |
| Unknown | 10 | - | - |  |  |  |
| Disease status‡ |  |  |  |  |  |  |
| Refractory | 46 | 13 | 28.3 | Reference |  |  |
| Relapsed | 53 | 10 | 18.9 | 0.590 | 0.230, 1.513 | 0.272 |
| Unknown | 5 | - | - |  |  |  |
| CD25 status |  |  |  |  |  |  |
| Positive | 28 | 7 | 25.0 | Reference |  |  |
| Negative | 24 | 3 | 12.5 | 0.429 | 0.097, 1.886 | 0.262 |
| Unknown | 52 | 13 | 25.0 |  |  |  |
| CD30 status |  |  |  |  |  |  |
| Positive | 40 | 9 | 22.5 | Reference |  |  |
| Negative | 44 | 9 | 20.5 | 0.886 | 0.312, 2.513 | 0.820 |
| Unknown | 20 | 5 | 25.0 |  |  |  |
| ECOG-PS |  |  |  |  |  |  |
| 0 | 14 | 8 | 57.1 |  |  |  |
| 1 | 42 | 9 | 21.4 |  |  |  |
| 2 | 26 | 5 | 19.2 |  |  |  |
| 3 | 14 | - | - |  |  |  |
| 4 | 8 | - | - |  |  |  |
| ECOG-PS |  |  |  |  |  |  |
| 0 or 1 | 56 | 17 | 30.4 | Reference |  |  |
| ≥2 | 48 | 6 | 12.5 | 0.328 | 0.117, 0.916 | 0.033 |
| Number of prior chemotherapy lines |  |  |  |  |  |  |
| 1 | 13 | 2 | 15.4 |  |  |  |
| 2 | 19 | 3 | 15.8 |  |  |  |
| 3 | 20 | 6 | 30.0 |  |  |  |
| 4 | 18 | 5 | 27.8 |  |  |  |
| 5 | 19 | 2 | 10.5 |  |  |  |
| ≥6 | 15 | 5 | 33.3 |  |  |  |
| Number of prior chemotherapy lines |  |  |  |  |  |  |
| ≤2 | 32 | 5 | 15.6 | Reference |  |  |
| ≥3 | 72 | 18 | 25.0 | 1.800 | 0.603, 5.371 | 0.292 |
| Combination Therapy |  |  |  |  |  |  |
| No | 89 | 20 | 22.5 | Reference |  |  |
| Yes | 15 | 3 | 20.0 | 0.863 | 0.221, 3.359 | 0.831 |
| Preventive drugs for ADRs |  |  |  |  |  |  |
| No | 22 | 7 | 31.8 | Reference |  |  |
| Yes | 82 | 16 | 19.5 | 0.519 | 0.182, 1.485 | 0.222 |
| Prior hematopoietic stem cell transplantation |  |  |  |  |  |  |
| No | 96 | 22 | 22.9 | Reference |  |  |
| Yes | 8 | - | - | 0.481 | 0.056, 4.120 | 0.504 |
| LDH |  |  |  |  |  |  |
| <223 U/L | 25 | 11 | 44.0 | Reference |  |  |
| ≥223 U/L | 77 | 11 | 14.3 | 0.212 | 0.077, 0.586 | 0.003 |
| Not measured | 2 | - | - |  |  |  |
| sIL-2R |  |  |  |  |  |  |
| <2000 U/mL | 29 | 8 | 27.6 | Reference |  |  |
| ≥2000 U/mL | 54 | 8 | 14.8 | 0.457 | 0.151, 1.382 | 0.165 |
| Not measured | 21 | 7 | 33.3 |  |  |  |

†Including patients with overlapping diagnoses.

‡Refractory disease was defined as a tumor evaluation of progressive disease after the most recent chemotherapy. Relapsed disease was defined as a tumor evaluation of complete response, partial response, or stable disease after the most recent chemotherapy. If no information on the most recent chemotherapy or tumor evaluation was available, or if the tumor evaluation was not evaluated or unknown, the patient’s disease status was categorized as unknown.

ADR, adverse drug reaction; CI, confidence interval; ECOG-PS, Eastern Cooperative Oncology Group Performance Status; LDH, lactate dehydrogenase; PTCL, peripheral T-cell lymphoma; sIL-2R, soluble interleukin-2 receptor; WHO, World Health Organization.
